# Near-field surface plasmons on quasicrystal metasurfaces

**DOI:** 10.1038/s41598-016-0027-y

**Published:** 2016-12-23

**Authors:** Quanlong Yang, Xueqian Zhang, Shaoxian Li, Quan Xu, Ranjan Singh, Yongmin Liu, Yanfeng Li, Sergey S. Kruk, Jianqiang Gu, Jiaguang Han, Weili Zhang

**Affiliations:** 1 Center for Terahertz waves and College of Precision Instrument and Optoelectronics Engineering, Tianjin University and the Key Laboratory of Optoelectronics Information and Technology (Ministry of Education), Tianjin, 300072 China; 2 Cooperative Innovation Center of Terahertz Science, Chengdu, 610054 China; 3 Center for Disruptive Photonic Technologies, Division of Physics and Applied Physics, School of Physical and Mathematical Sciences, Nanyang Technological University, 21 Nanyang, Link, 637371 Singapore; 4 Department of Mechanical and Industrial Engineering, Northeastern University, Boston, MA 02115 USA; 5 Nonlinear Physics Center and Center for Ultrahigh Bandwidth Devices for Optical Systems, Research School of Physics and Engineering, The Australian National University, Canberra, Australian Capital Territory 2601 Australia; 6 School of Electrical and Computer Engineering, Oklahoma State University, Stillwater, Oklahoma 74078 USA

## Abstract

Excitation and manipulation of surface plasmons (SPs) are essential in developing cutting-edge plasmonic devices for medical diagnostics, biochemical spectroscopy and communications. The most common approach involves designing an array of periodic slits or grating apertures that enables coupling of the incident light to the SP modes. In recent years, plasmonic resonances, including extraordinary optical transmission through periodic arrays, quasicrystals and random aperture arrays, have been investigated in the free space. However, most of the studies have been limited to the far field detection of the transmission resonance. Here, we perform near-field measurements of the SPs on quasicrystal metasurfaces. We discover that the reciprocal vector determines the propagation modes of the SPs in the quasicrystal lattice which can be well explained by the quasi-momentum conservation rule. Our findings demonstrate vast potential in developing plasmonic metasurfaces with unique device functionalities that are controlled by the propagation modes of the SPs in quasicrystals.

## Introduction

Surface plasmons (SPs) in periodic subwavelength aperture arrays have been widely investigated because of their potential applications in near-field imaging^1–5^, sensing^6^ and extraordinary optical transmission (EOT)^7–9^. Structural periodicity that provides additional momentum required in coupling to the SP modes is crucial in SP excitation^4^. Meanwhile, optical resonances including EOT and scattering properties have been realized by aperiodic arrays, such as quasicrystals or random subwavelength apertures^10–19^. The aperiodic arrays open a new avenue to explore the unique mechanism between SPs and lattice arrangements. Of particular interest are quasicrystal arrays which exhibit *n*-fold rotational symmetry. One-dimensional and two-dimensional photonic quasicrystal structures are intriguing since they have manifested exotic properties for a number of photonic applications such as negative refraction^20^, waveguiding^21^, imaging^22–25^, and optical modes^26^. These properties primarily rely on the presence of multiple band gaps and multi-frequency characters^27–34^, resulting from a large number of reciprocal vectors presented in the reciprocal lattice space^11,20,35,36^. A prevailing method in quasicrystal study originates from the implementation of quasicrystal metasurfaces (QCMs) that may replace bulky devices with ultrathin elements^17^. Previous works on QCMs have mainly focused on transmission, photonic band gaps and optical modes in the far field^37–41^. However, the behaviors of SPs in QCMs remain unexplored. In this work, we present the first demonstration of a near-field SP distribution of QCM that exhibits a directional propagation characteristic. In order to understand the unique properties of SPs in QCMs, here we also compare the SP response with metasurfaces based on two other fundamentally different distributions: *periodic* and *random* at terahertz frequencies. Our findings clearly reveal the role and impact of the structural periodicity on SP propagation properties supported by metasurfaces, opening a new avenue to manipulate SPs.

For a QCM with rotational symmetry, the reciprocal vectors (**G**_*i*_) in the Fourier space have a direct relationship with the wave vector of the allowed SPs^10^. It has been shown that QCMs with slit arrays exhibit resonances governed by a quasi-momentum rule given by: **k**_//_ + **G**_*i*_ = **k**_sp_, where **k**_//_ is the light wave vector parallel to the metasurface plane, ***k***_sp_ is the wave vector of SPs, and **G**_*i*_ is a reciprocal vector that can be replaced by the Fourier transform vectors since QCMs exhibit a long-range order. It is obvious that **k**_sp_ = **G**_*i*_ for normal incidence. Thus, the wave vector **k**_sp_ depends on the distributions of the slit arrays, and SPs excited from the QCMs could propagate in the directions that correspond to all the involved reciprocal vectors **G**_*i*_.

To confirm the preceding prediction based on the quasi-momentum conservation rule, we calculated and examined the propagation of the SPs on metallic QCM that possess 8-fold and 10-fold rotational symmetry. Furthermore, a theoretical model based on the Huygens-Fresnel principle is employed to analyze the prediction^42^. We show that the reciprocal vectors **G**_*i*_ control the excitation and propagation of the SP resonances. This is also the first demonstration of a near-field SP distribution of QCM. It is found that the propagation modes of the SPs on 8-fold QCM exhibit an identical rule of reciprocal vectors. This prediction can be applied to QCMs with different order rotational symmetry. For comparison, the SP distributions of periodic and random aperture arrays are also investigated. The unique properties of QCMs offer a new degree of freedom for excitation, control and propagation of SPs.

## Results

### Sample diagram and reciprocal space of 8-fold QCM

**Figure 1:**
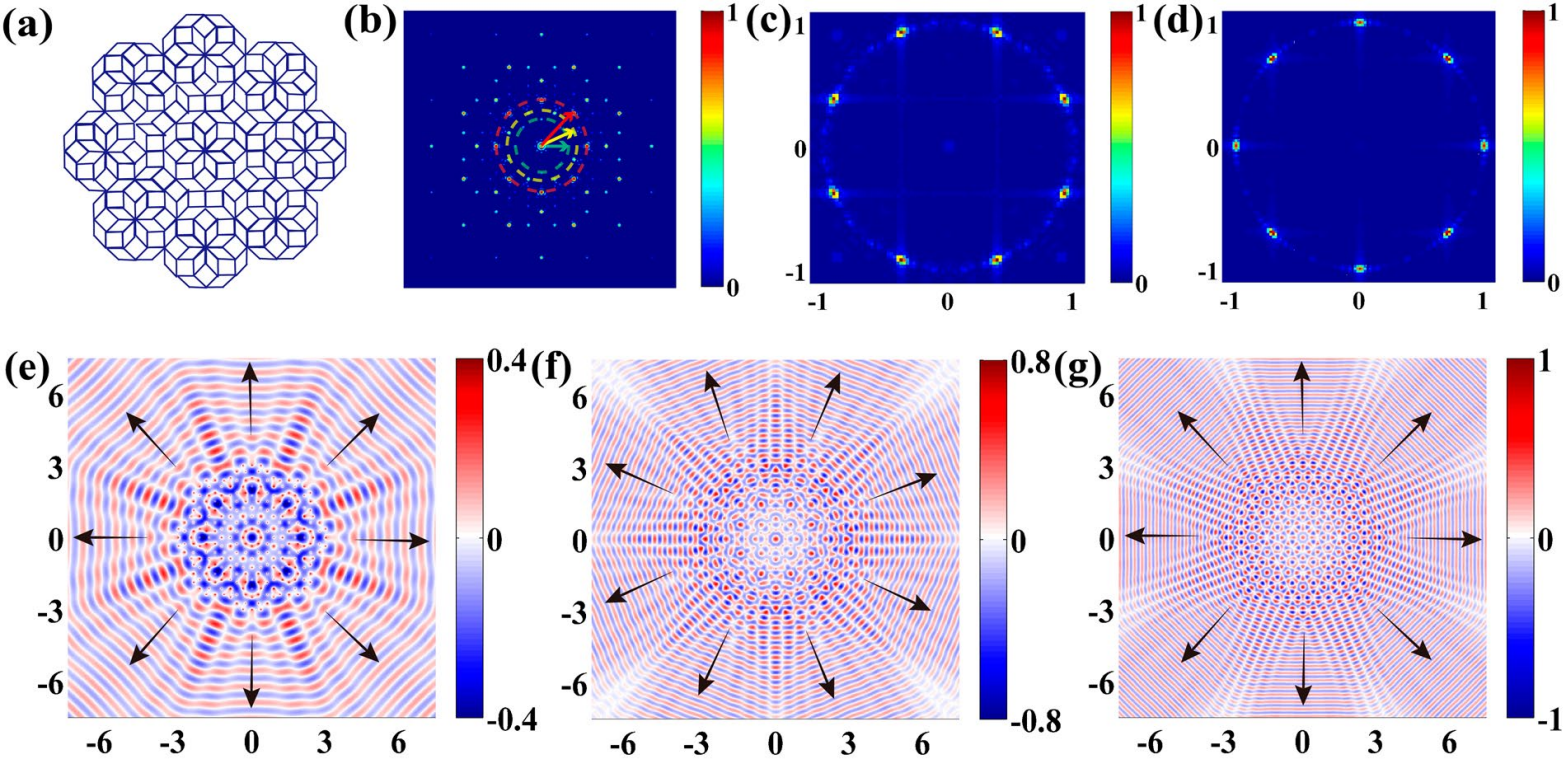
Theoretical studies of 8-fold rotationally symmetric QCM. (**a**) 2D theoretical model of 8-fold rotational symmetry quasicrystal apertures. (**b**) Reciprocal space of the quasicrystal in **a** calculated by 2D FFT, three circles with different colors define three different characteristic frequencies. Only one Fourier transform vectors for each frequency is marked by an arrow. (**c**) Frequency space calculated by Fourier decomposition at 0.78 THz. The coordinates of peaks define the angle of the propagation mode. (**d**) Corresponding frequency space at 0.58 and 1 THz. (**e,f,g**) Calculated SP distributions at three characteristic frequencies of 0.58 (**e**), 0.78 (**f**), and 1 THz (**g**). The arrows define the propagation directions of the SP plane waves. The unit is **mm**.

Figure 1a shows the schematic of an 8-fold rotationally symmetric QCM, where the tiles are based on two types of rhombus: a thin tile with vertex angles of 45° and 135° and a square tile marked by the blue line^43^. The equal length of the rhombus side is defined as *P*. The illustrated 2D patterned area contains 264 points, located at the vertices of each rhombus. Signatures of the SP propagation in 8-fold QCM can be found in the reciprocal lattice space calculated by 2D Fast Fourier Transform (FFT), as shown in Fig. 1b. The pattern also possesses an 8-fold rotational symmetry, and contains several spots with different intensities. The positions of diffraction peak that appear in the Fourier transform of QCM are related to the length of the rhombus side in the real space. The peaks on the same circle denote the directions of the Fourier transform vectors at the same frequency which are called reciprocal vectors^10^ (see the arrows in Fig. 1b). QCMs hold infinite number of discrete characteristic frequencies. In this work, we only focus on three frequencies below 1 THz. The fundamental characteristics of the reciprocal vectors at other frequencies can be captured by these frequencies.

For a given *P* = 365 μm, we chose three characteristic frequencies: 0.58, 0.78, and 1 THz (Supplementary Fig. 1 and Note 1). In Fig. 1(b), we used dashed circles of different colors to mark these characteristic frequencies. Meanwhile, according to the reciprocal vectors of the characteristic frequencies at 0.58 and 1 THz, there are eight specific angles of the reciprocal vectors defined relatively to the positive horizontal direction: {0°, 45°, 90°, 135°, 180°, 225°, 270°, 315°}; while at 0.78 THz, they are {22.5°, 67.5°, 112.5°, 157.5°, 202.5°, 247.5°, 292.5°, 337.5°}. These angles result from the 8-fold rotational symmetry determines the wave vectors and thus the propagation directions of the SPs.

### Theoretical analysis

We calculated the distribution of a monochromatic surface field ${\tilde{E}}_{z}^{sp}$ along a planar surface by the Huygens-Fresnel principle to examine the prediction:

1 $${\tilde{E}}_{z}^{sp}(x,y)=\sum _{{\rm{j}}}-\frac{i}{\sqrt{{\lambda }_{sp}}}\frac{{e}^{i{k}_{sp}r}}{r}\,\cos \,\theta {E}_{0}^{sp}({x}_{j},{y}_{j}){e}^{i\frac{\pi }{4}},$$

where ${E}_{0}^{sp}({x}_{j},{y}_{j})$ is amplitude of each point source, *λ*_*sp*_ = 2*π*/*k*_*sp*_, *x*_j_ and *y*_j_ represent the coordinates of the point source, $r=\sqrt{{(x-{x}_{j})}^{2}+{(y-{y}_{j})}^{2}}$ and *θ* = arc cos(*x*/*r*). To excite the SPs, each point at the vertices of the rhombus is taken as a perfect point source (cos*θ* = 1) in this model, which determines the SP distribution at the planar surface as given by the above formula.

In the *x-y* plane at a height of 50 μm above the upper surface of the structure, we theoretically calculated the electric field amplitude *E*_*z*_ at three characteristic frequencies shown in Fig. 1e–g. It is observed that the SPs propagate in the specific directions defined by their respective reciprocal vectors at all the three characteristic frequencies, agreeing well with our prediction. We define these SPs propagating outward from the excitation region as the propagation modes at the characteristic frequencies. In order to observe the fields clearly, we normalized the amplitude of the electric field at each characteristic frequency and tuned the color bar proportionately. Figure 1e,g show the SP field distributions at 0.58 and 1 THz, respectively. There are eight SP plane waves along the respective angles of {0°, 45°, 90°, 135°, 180°, 225°, 270°, 315°}. Each specific angle of the propagation modes requires detailed analysis. To clearly distinguish each propagation mode, arrows are added in the figure for clear visualization. Additionally, there are also eight propagation modes as predicted theoretically at 0.78 THz in Fig. 1f. The propagation modes at 0.78 THz are different from those at the other two frequencies due to different reciprocal vector directions. It is interesting to note that several fringes are excited between two propagation modes mainly due to the interference of two adjacent modes, which highlights the coherence of each propagation mode. At higher characteristic frequencies, we observe the formation of a larger number of fringes.

Here, we obtain the propagation properties of the SP waves qualitatively. From Fig. 1e–g, the SP field distributions could be assumed to be a linear superposition of plane waves propagating along different directions. We use the Fourier decomposition to further understand this. The complex amplitude of the field distribution can be written as:

2 $${\bf{U}}(x,y)=\sum _{i}{{\bf{U}}}_{i}=\sum _{i}{A}_{i}\,\exp [i\frac{2\pi }{\lambda }(x\,\cos \,{\alpha }_{i}+y\,\cos \,{\beta }_{i})],$$

where *A*_*i*_ is the amplitude of each SP wave, cos *α*_*i*_ and cos *β*_*i*_ are the direction cosines of the SP wave vector, and the space frequencies are *f*_*x*_ = cos*α*_*i*_/*λ*, and *f*_*y*_ = cos*β*_*i*_/*λ*. For a monochromatic SP wave, we multiply the wavelength and space frequency, and then cos *α*_*i*_ and cos *β*_*i*_ represent the coordinates of the corresponding amplitude in the frequency space, and the numerical values of cos *α*_*i*_ and cos *β*_*i*_ in the frequency space determine the propagation angles of the SP waves.

Figure 1c,d show the frequency space that calculated by the Fourier decomposition. As shown in Fig. 1c, there are eight propagation modes with propagation angles of {0°, 44.8°, 89.8°, 135.2°, 180°, 224.8°, 269.8°, 315.2°} at 0.58 and 1 THz, which is consistent with the previously calculated SP field of the 8-fold QCM. Meanwhile, the number of corresponding propagation modes at 0.78 THz is also eight, as shown in Fig. 1d, where the calculated propagation angles are {22.6°, 67.4°, 112.6°, 157.4°, 202.6°, 247.4°, 292.6°, 37.4°}. The slight discrepancy (±0.1° at 0.78 THz, ±0.2° for others) between the calculated and ideal angles is due to limited spectral resolution.

### Numerical simulations and experimental verification

**Figure 2:**
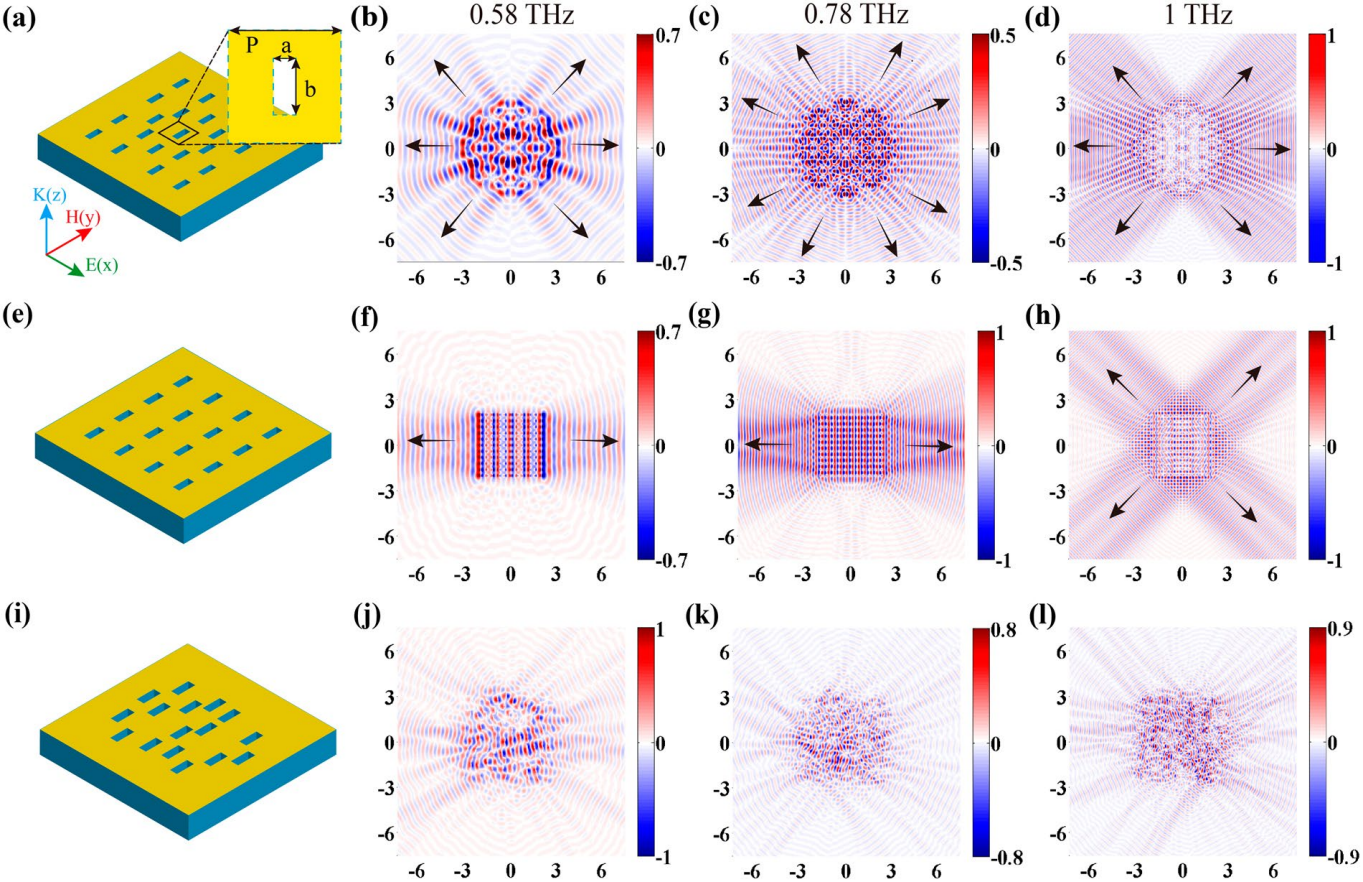
Simulated field distributions of QCM, PAM and RAM. Schematic diagram of 8-fold QCM (**a**), PAM (**e**) and RAM (**i**) with slits patterned on metallic films. Inset of (**a**): enlarged single slit with the width of a = 60 μm and length of b = 150 μm. (**b–d**) Simulated SP distributions of the 8-fold QCM at frequencies of 0.58 (**b**), 0.78 (**c**), and 1 THz (**d**). (**f–h**) Simulated SP distributions of PAM at 0.58, 0.78, and 1 THz, respectively. (**j–l**) Corresponding SP distributions of RAM at three frequencies, respectively.

To validate the calculation, we compare three structures with different symmetries. The first sample is a metasurface that consists of slits arranged into 8-fold rotationally symmetric layout as shown in Fig. 2a, the second is a metasurface with periodically arranged slits (PAM) in Fig. 2e, and the last is a metasurface with randomly arranged slits (RAM) in Fig. 2i. The structures were designed to have the same amount of elements, and the period of PAM is equal to the side length of QCM. Polarization of the incident THz wave is perpendicular to the longer side of the slits. The simulation results of the 8-fold QCM are given in Fig. 2b–d, where the electric field distribution of SPs at the characteristic frequencies 0.58, 0.78 and 1 THz are illustrated. The simulation results are consistent with theoretical prediction at 0.78THz. At other frequencies, the propagation modes along the angles {0°, 45°, 135°, 180°, 225°, 315°} also show the same characteristic as predicted by the theory (Supplementary Fig. 2). However, the propagation modes in the vertical direction disappear. These differences can be explored when a single SP source is taken into consideration. The SPs in a slit acts as a dipole source under linear polarization excitation, which is significantly different from a perfect point source that we used in Eq. (1). The mismatch between the calculated and simulated results can be attributed to magnetic dipoles induced by the slits do not radiate in the direction that perpendicular to the incident polarization^44^ (Supplementary Fig. 3). Another noteworthy observation is that the propagation modes close to horizontal polarization direction have larger amplitude. This could be described by using the relation between the intensity in slits and the azimuth angle (Supplementary Table 1).

For comparison, the SP distributions in PAM and RAM are presented. Figure 2f–h show the SP propagation in PAM at three chosen frequencies. As we know, the reciprocal lattice space of PAM is still periodic, the basic characteristic frequency can be calculated by two adjacent diffraction peaks, and other higher characteristic frequencies can be deduced from the fundamental characteristic frequency^7,45^. Here, only the SP field distributions at 0.58, 0.78 and 1 THz are given for comparison. At 0.58 and 0.78 THz, the SP field reveals a bidirectional plane wave propagation behavior in the horizontal direction under linear polarization incidence. However, the SP with a plane wave behavior propagates along the diagonal direction at 1 THz, where the corresponding propagation modes with associated wave vectors along the diagonal of the period are excited. Figure 2j–l show the SP distributions in RAM, where there is no propagation of regular SPs from the excitation region since the reciprocal lattice space does not have a consistent pattern of either periodicity or rotational symmetry.

**Figure 3:**
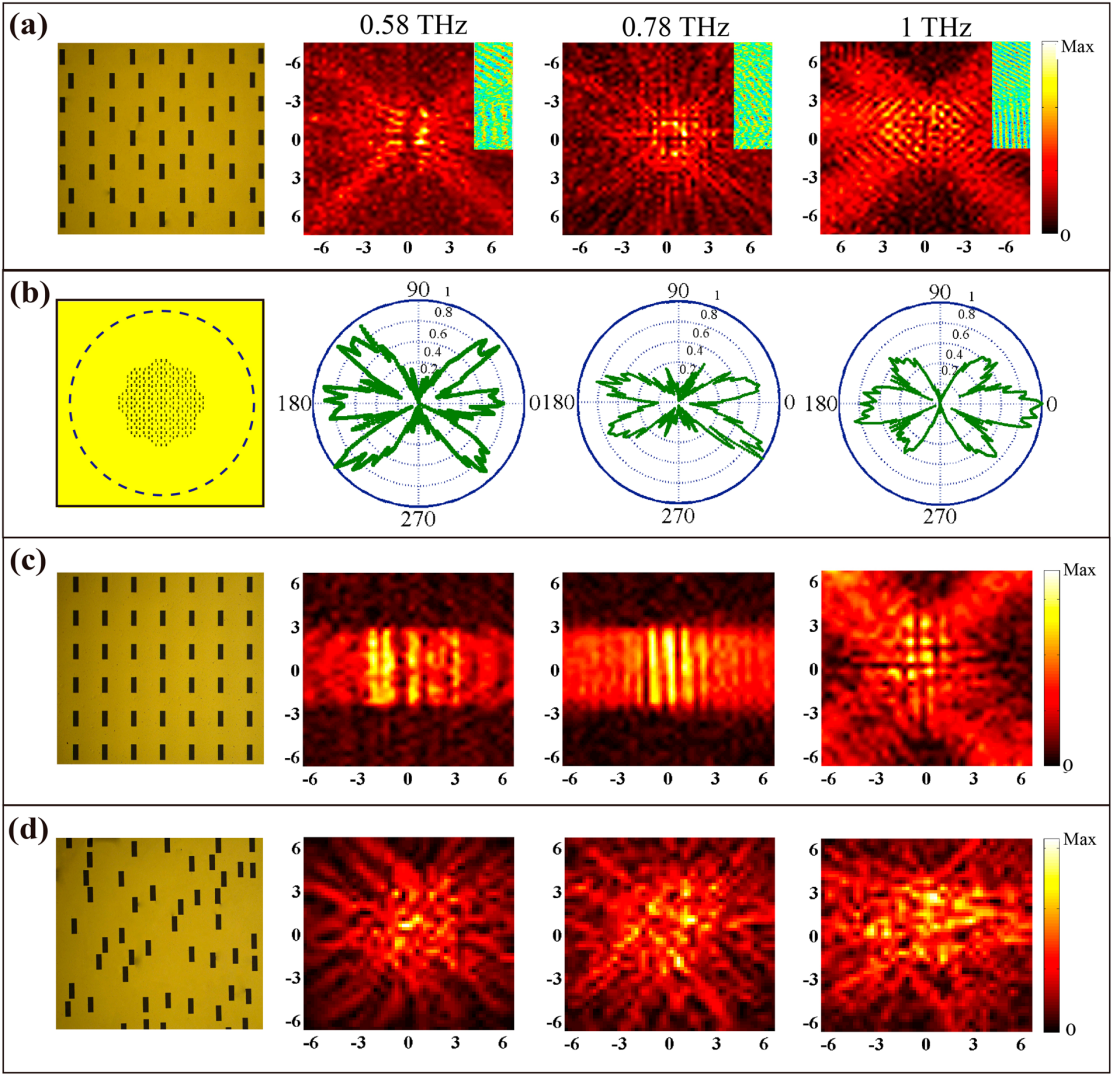
Measured SP intensity distributions. (**a**) Measured electric intensity of the 8-fold QCM at 0.58 (left), 0.78 (center) and 1THz (right) under linear polarization excitation. Insets of (**a**): high-resolution distributions of the measured real-part *E*_*z*_-field at the same place where measured electric intensity is occupyed. (**b**) Angle-resolved electric field distributions of the SP waves scanning along the dashed blue circle line with a radius r = 10 mm. (**c,d**) Corresponding measured SPs intensity of the PAM (**b**) and RAM (**c**) at three characteristic frequencies.

To experimentally confirm the phenomena in the metasurfaces, we fabricated three different types of samples consisting of an 8-fold QCM, a PAM and a RAM with the same geometric parameters as those used in the simulations by conventional photolithography. We measured the electric field distribution on three metasurfaces at 0.58, 0.78 and 1 THz under linear horizontal polarization excitation, as shown in Fig. 3. We notice that there are six propagation modes along the predicted angles, when at 0.58 (left) and 1 THz (right), and the number of measured propagation modes at 0.78 THz (center) is eight in Fig. 3a. Moreover, it is also particularly evident that the measured real-part SPs *E*_*z*_-field in the inset of Fig. 3a propagates as a plane wave along the predicted angle. The agreements between the simulated and measured results are shown in Figs 2 and 3a. In addition, the angle-dependent electric distributions of the SP waves are also discussed. As shown in Fig. 3b, we scanned the electric field of the SP waves along the dashed circle line with a radius *r* = 10 mm. We found that the angles of maximum amplitude of the SP waves are in good agreement with the former simulated and measured results. We further carried out the measurements for the PAM and RAM samples, as shown in Fig. 3c,d. In comparison to Fig. 2, we observe that the SP distributions have a good agreement with the simulation results.

## Discussion

**Figure 4:**
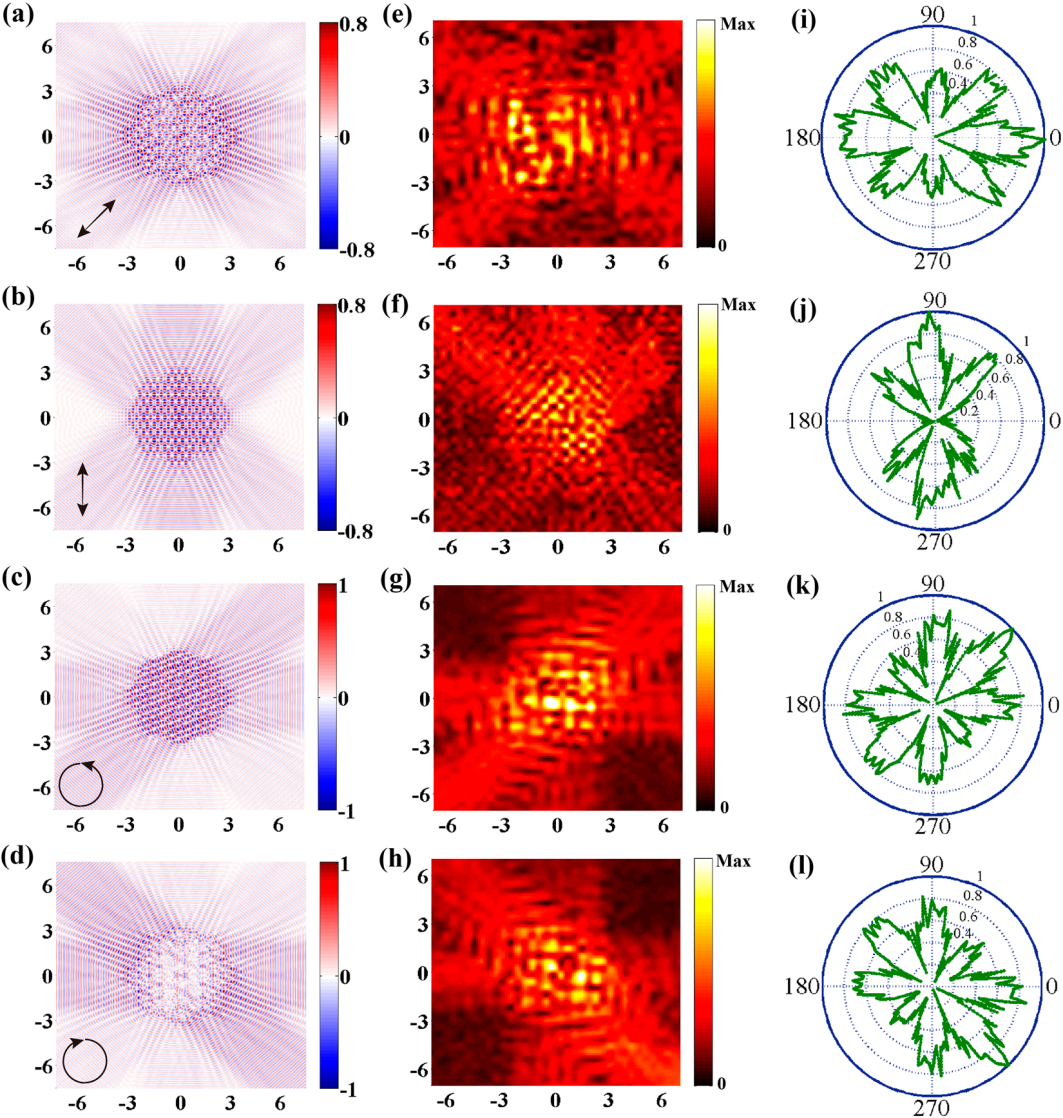
Simulated and mesured SPs of QCM with different polarizations. (**a–d**) Simulated SP distributions of the 8-fold QCM at 1 THz under normally incident terahertz waves with 45° polarization direction (**a**), vertical polarization (**b**), LCP (**c**) and RCP (**d**). (**e–h**) Corresponding measured SP intensity at 1THz under four polarizations, respectively. (**i–l**) Angle-resolved electric filed distributions of the SPs mode at 1.0 THz for different polarizations incidence, respectively.

Furthermore, the SP propagation in the proposed 8-fold QCM has a strong dependence on polarization. Figure 4a,e shows the simulated (left) and measured (right) SP field distribution at 1 THz when excited with a linearly polarized light at an angle of 45°, there are eight SP plane waves propagate along the expected angles, but the SP field along the vertical directions are weaker. For vertical polarization excitation, only six propagation modes could be observed without the horizontal direction, as illustrated in Fig. 4b,f, which is on the contrary to the case of horizontal polarization excitation. These phenomena depend strongly on the SP field distribution in individual slit (Supplementary Figs 2 and 3). Figure 4c,d,g,h show the propagation mode distributions for the left-handed circular polarization (LCP) and right-handed circular polarization (RCP) excitations, respectively. One important feature of the circular polarization excitation is that the SP field in single slit can be excited in all directions. However, the strength of the field would be quite different due to the slit geometry. The amplitude of the SP field *E*_*z*_ for horizontal and 45° are greater than other propagation modes for the LCP excitation. However, for the RCP excitation, the corresponding propagation modes fall in the horizontal and 135° direction (Supplementary Table. 1). It can therefore be deduced that the largest *E*_*z*_ of a specific propagation mode for RCP and LCP excitation is based on a linear combination of the corresponding field distribution from the slits on the planar metasurface (Supplementary Fig. 3). Moreover, the angle-dependent electric field distributions of the SP waves at 1.0 THz for four polarizations are also measured, which show the same phenomenon with the simulated and measured results, as shown in Fig. 4i–l.

**Figure 5:**
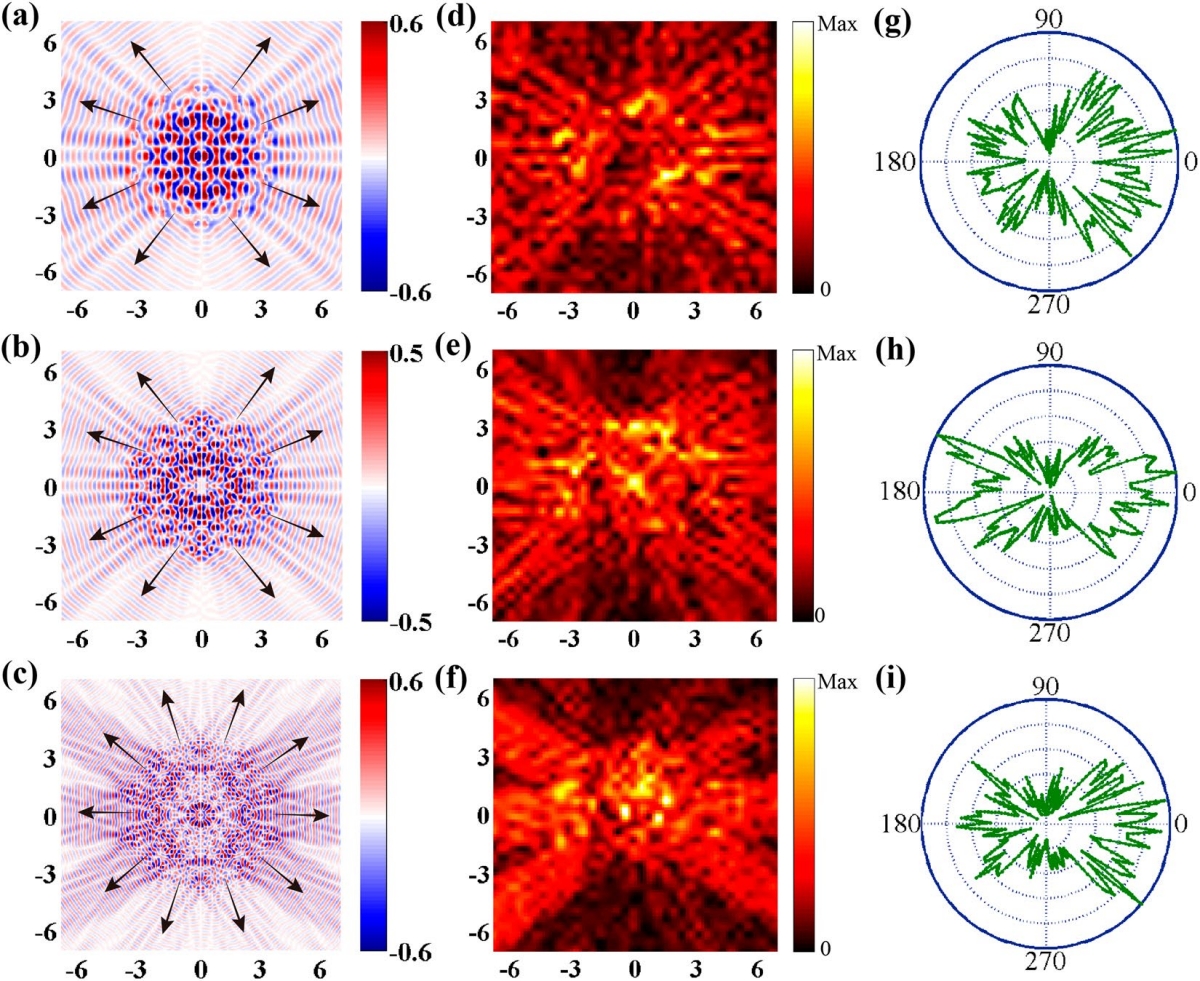
Simulated and mesured SPs distributions of the10-fold QCM. (**a–c**) Simulated SP distributions on the 10-fold QCM at 0.53, 0.7 and 1.02 THz under linear horizontal polarization excitation. (**d–f**) Corresponding measured electric intensity. (**g–i**) Angle-resolved electric field distributions of the SP waves.

To further verify that the analysis of the SP propagation behavior in the 8-fold QCM is applicable to QCMs with different rotational symmetries, we calculated and measured the SP field distributions in a 10-fold QCM, as shown in Fig. 5. The characteristic frequencies of the 10-fold QCM can be calculated and chosen for the given *P* = 365 μm as 0.53, 0.7 and 1.02 THz. Besides, from the simulated 2D electric fields and measured electric intensity of SPs at these characteristic frequencies in Fig. 5a–f, there are ten propagation modes along specific angles of {0°, 36°, 72°, 108°, 144°, 180°, 216°, 252°, 288°, 324°} at 1.02 THz. However, only eight propagation modes along angles of {18°, 54°, 126°, 162°, 198°, 234°, 306°, 342°} are observed at 0.53 and 0.7 THz. Figure 5g–i show the angle-resolved electric field distributions of the SP waves of the 10-fold QCM. Similar to the phenomena of the 8-fold QCM, the propagation modes of the 10-fold QCM becomes weaker as the angle with respect to the horizontal direction increases. This can also be related to the SPs excited in the single slit by the horizontal linearly polarized light. It is obvious that the nature of the propagation modes in the 10-fold QCM has a field distribution that follows a similar trend as in that of the 8-fold QCM. This further validates our prediction. It is thus expected that the propagation modes of higher order rotationally symmetric QCMs would possess the same characteristic as the 8-fold QCM. However, it is worth noting that with higher order rotational symmetry, the fringes excited between the two modes increases, which makes it difficult to distinguish between each propagation mode if the observation area is near to the excitation region.

The preceding analysis shows that the SP of the slits on metallic QCM will propagate in the directions of **G**_*i*_ under the condition of normal incidence. As per our discussion in the previous section, we conclude that the propagation behaviors of the SPs in QCMs are determined by two parts. First, permutation and combination of slits constitute a group that results in a specific propagation behavior of the SPs at discrete characteristic frequencies. This leads to a big disparity between the SP propagation in QCMs and PAMs. In addition, the excitation polarization has a large influence on the field intensity distribution in QCMs, which determines the amplitude of propagation mode that could vary from a weak existence to a strong existence, without any noticeable change in the propagation behavior of the SPs.

In summary, propagation properties of SPs in QCMs are demonstrated using analytical theory and near-field measurements. Due to rotational symmetry of QCMs, propagation of the SPs exhibits distinct characteristics that are fundamentally different from that of periodically and randomly arranged metasurfaces. This unique approach paves the way towards promising applications in the excitation and control of near-field SPs. Plasmonic mechanism of QCMs observed in the terahertz regime could also be extended to a broader spectrum of electromagnetic waves.

## Methods

### Simulations

Numerical simulations were carried out using the finite-element time-domain solver of *CST Microwave Studio*. The entire simulation area was 15 mm × 15 mm. All slits with the width of *a* = 60 μm and height of *b* = 150 μm for three different patterns were fabricated on 200 nm-thickness free standing aluminum foils. To support the metallic foils, the metasurface samples were designed as a sandwiched structure between bi-layer polyimide with a permittivity of *ε* = 2.93 and a loss tangent of *δ* = 0.044 at 1 THz (measured by experiment). Each polyimide layer has a thickness of 10 μm. The structure consists of 264 unit cells and was located at the center of the simulation area. Open boundary conditions were applied in both the *x* and *y* directions. The incident wave was *x*-polarized and illuminated on the metasurface at normal incidence from the substrate side. Field distributions of the SPs were mapped by defining the electric field monitors at 0.58, 0.78 and 1 THz. The simulation results were obtained at 50 µm above the upper surface of the metasurface devices.

### Experiments

The electric field component *E*_*z*_ of the SPs was detected by a near-field scanning terahertz microscopy system, where the entire excitation area of the samples with a diameter of 5 mm was covered by a nearly uniform linearly polarized terahertz beam at normal incidence. A polarizer was placed in front of the samples to control the polarization direction of the terahertz field. A fiber-coupled terahertz near-field probe with a resolution of 20 μm was used as the detector and was mounted on a two dimensional translation stage to enable 2D scans at a fixed distance from the sample surface. The 2D electric field was detected in two modes: the fast mode with 0.25 mm per step in both the *x* and *y* directions from −7 mm to +7 mm; the precise mode with 100 μm per step in the *y* direction from 0 mm to 7 mm and in the *x* direction from 5 mm to 7 mm. With the advantages of the time-domain measurement of near-field SPs, both the amplitude and phase of SPs at desired frequencies can be obtained by FFT, which is extremely important to map the SPs field.

## Electronic supplementary material

Supplementary Information
