# Supplementary material for: Near-field surface plasmons on quasicrystal metasurfaces
Source: Sci Rep. 2016 Dec 23;6:26. doi: 10.1038/s41598-016-0027-y (PMC5431347; doi:10.1038/s41598-016-0027-y)
Supplement: Supplementary file 1 — Supplementary Information [file 41598_2016_27_MOESM1_ESM.pdf]

Supplementary Information

Near-field surface plasmons on quasicrystal metasurfaces

Quanlong Yang,[1,2] Xueqian Zhang,[1,2] Shaoxian Li,[1,2] Quan Xu,[1,2] Ranjan Singh,[3] Yongmin Liu,[4] Yanfeng Li,[1,2] Sergey S. Kruk[5], Jianqiang Gu,[1,2] Jiaguang Han,[1,2] Weili Zhang[1,2,6]

[1]Center for Terahertz waves and College of Precision Instrument and Optoelectronics Engineering, Tianjin University and the Key Laboratory of Optoelectronics Information and Technology (Ministry of Education), Tianjin 300072, China

[2]Cooperative Innovation Center of Terahertz Science, Chengdu 610054, China

[3]Center for Disruptive Photonic Technologies, Division of Physics and Applied Physics, School of Physical and Mathematical Sciences, Nanyang Technological University, 21 Nanyang Link 637371, Singapore

[4]Department of Mechanical and Industrial Engineering, Northeastern University, Boston, MA 02115, USA

[5]Nonlinear Physics Center and Center for Ultrahigh Bandwidth Devices for Optical Systems, Research School of Physics and Engineering, The Australian National University, Canberra, Australian Capital Territory 2601, Australia

[6]School of Electrical and Computer Engineering, Oklahoma State University, Stillwater, Oklahoma 74078, USA

Correspondence and requests for materials should be addressed to J. H. (jiaghan@tju.edu.cn) or W. Z. (weili.zhang@okstate.edu)

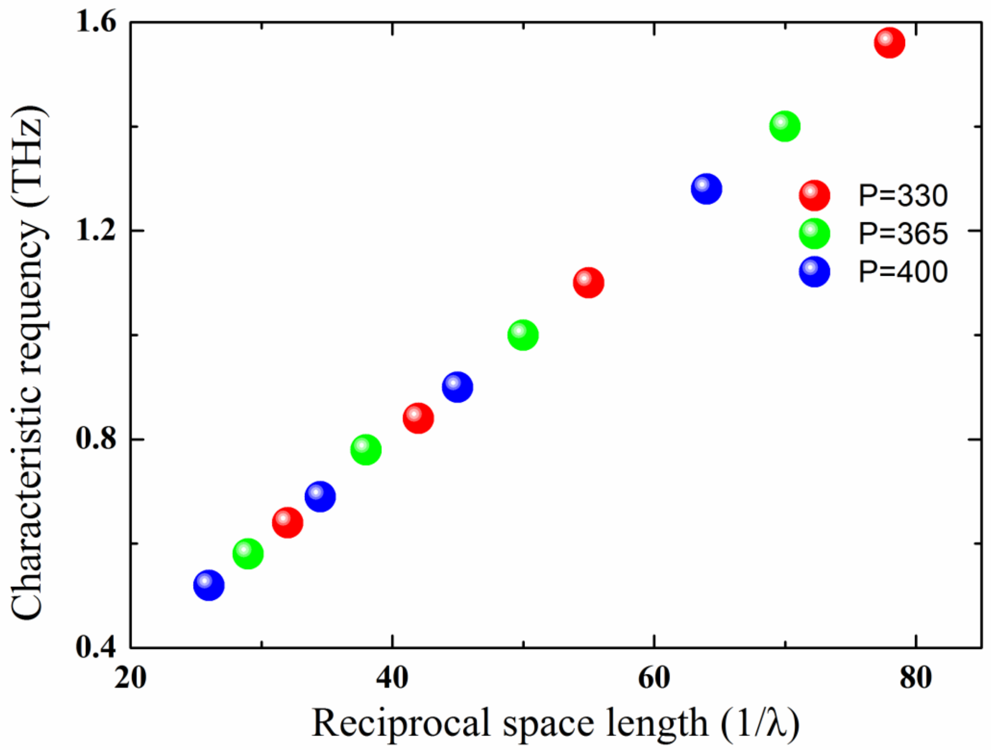

Supplementary Figure 1. Relations between characteristic frequencies and *P*. Relationship between characteristic frequencies and reciprocal space lengths (between the diffraction peaks and center point) are calculated: {0.58 (29), 0.78 (38), 1 (50) and 1.4 (70)}. Based on the same method, characteristic frequencies for both *P* = 330 and 400 μm are also calculated: {0.64 (32), 0.84 (42), 1.1 (55), 1.56 (78)} and {0.52 (26), 0.69 (34.5), 0.9 (45), 1.28 (64)}. These indicate that the characteristic frequencies are proportional to the radius of the circles shown in Figs. 1b that are inversely proportional to the length of the rhombus side in the real space. When the *P* changes, the corresponding characteristic frequencies shift in the opposite way, but the linear relationship between them remains constant.

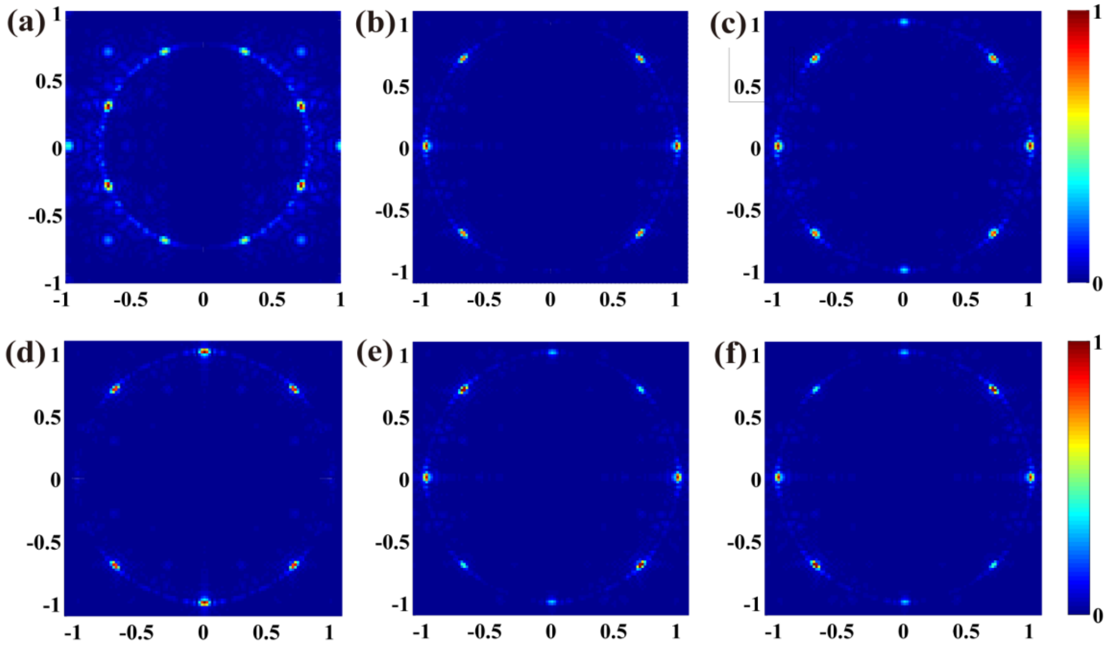

Supplementary Figure 2. Frequency space of simulated SPs. (**a**) Frequency space of simulated SPs in the case of linear horizontal polarization at 0.78 THz. The peak points denote the corresponding propagation modes (PMs), and their intensity represents the amplitude of the propagation modes. It is obvious that the number of PMs at 0.78 THz is eight. However, there are many additional peak points at 0.78 THz. In this case, a large number of fringes are excited between two propagation modes causing the interference of two adjacent propagation modes, and the azimuthal angle of these points are the same as the fringes, and they denote the propagation mode of the fringes. (**b**) Frequency space of the simulated SPs with linear horizontal polarization at 0.58 and 1 THz, where the corresponding PMs are six in number. (**c, d**) Frequency spaces of linear polarization along the directions of 45° and 90°, respectively. The distributions of peak-points are consistent with simulated terahertz SPs in all polarization directions. (**e, f**) Corresponding frequency spaces of RCP and LCP, respectively.

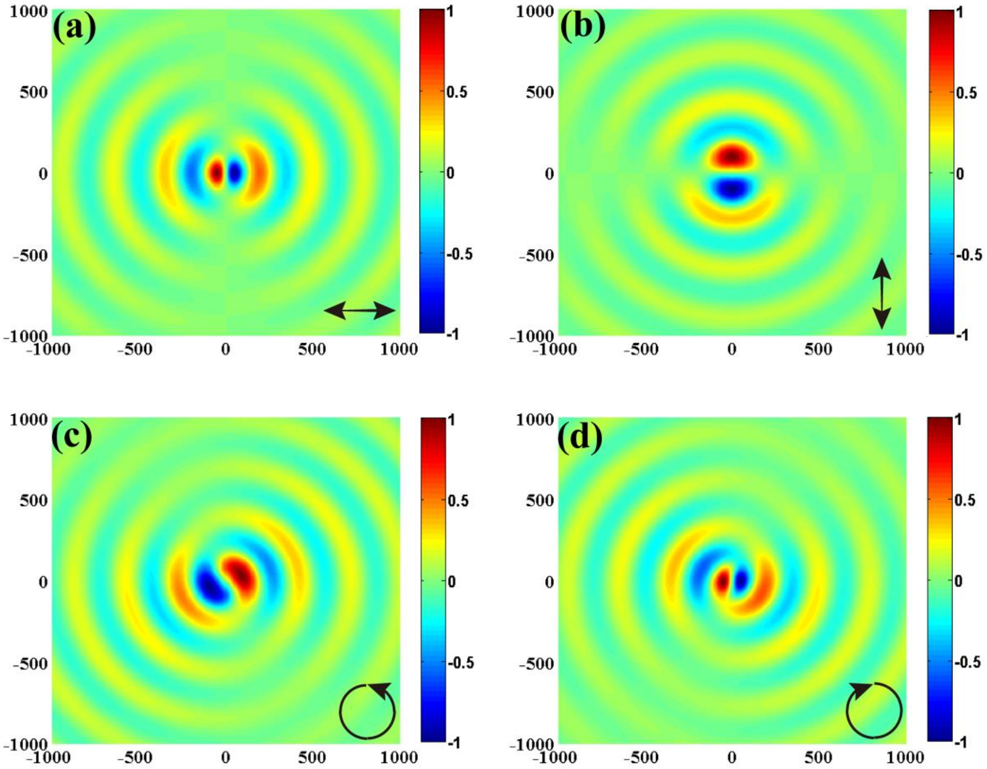

Supplementary Figure 3. SP distribution of single silt. (**a**) Simulated field distribution of single silt under horizontal polarization at 1 THz. In an ideal model, a point source radiates a spherical wave, and the electric amplitude of the SP wave is equal in all directions. It is obvious that the theoretically calculated SP amplitudes of the 8-fold quasicrystal are equal in all propagation modes. In reality, a slit under terahertz excitation with both vertical and horizontal linear polarizations can only be seen as a dipole source, and the obliquity factor should be taken into consideration, which determines the amplitude of the propagation modes in different polarization directions. In the case of horizontal polarization, E_z of the silt does not exist in the vertical polarization excitation. (**b**) E_z exists on the contrary in the vertical polarization excitation. Under linear polarization at 45°, it amounts to a superposition of the horizontal and vertical polarizations. With an aspect ratio of 2:5, the dipole features of a single slit can be excited under both horizontal and vertical polarizations, but two dipoles have different strengths. (**c, d**) Corresponding SP distributions of the RCP and LCP excitation. It is found that the intense amplitude of the SP field is along the angle of 22.5° for RCP and 157.5° for LCP, which is just located in the middle of two largest propagation modes. Therefore the larger amplitude along the specific propagation modes for RCP and LCP is based on the linear combination of the corresponding field distributions of the slits in the plane.

Characteristic Frequencies (THz)	0°	45°	90°	135°	180°	225°	270°	315°
**0.58(0°)**	1	0.65	0	0.65	1	0.65	0	0.65
**1(0°)**	1	0.74	0	0.74	1	0.74	0	0.74
**1(45°)**	1	0.79	0.37	0.79	1	0.79	0.37	0.79
**1(90°)**	0	0.82	1	0.82	0	0.82	1	0.82
**1(LCP)**	0.92	1	0.33	0.40	0.92	1	0.33	0.40
**1(RCP)**	0.92	0.41	0.29	1	0.92	0.41	0.29	1
	**22.5°**	**67.5°**	**112.5°**	**157.5°**	**202.5°**	**247.5°**	**292.5°**	**337.5°**
**0.78(0°)**	1	0.56	0.56	1	1	0.56	0.56	1

Supplementary Table 1. Amplitudes of PMs in different polarizations. The table shows the relationship between specific angle of reciprocal vector and the field amplitude from the data of Fourier decomposition. For clarity, we normalized the respective amplitude of the SPs. It should be noted that perfect alignment with horizontal direction that leads to the largest amplitude of the PMs is in the horizontal polarization. The amplitude of the SPs field (E_z) along the horizontal direction and 45°are greater than others for RCP, For LCP, however, the corresponding stronger propagation modes are along the horizontal direction and 135°, as can be seen in supplementary Figure. 3. This can be described by applying the relation between the field amplitude of single silt and the azimuthal angle. These data show the same trend as that of the SPs distributions.

Supplementary Note 1. Rule of characteristic frequencies and *P*

The rule between the characteristic frequencies and *P* is calculated to verify the theoretical SPs distributions of the 8-fold QCM. Diffraction peaks in the reciprocal space denote different reciprocal vectors, and their lengths to the central point are related to the characteristic frequencies. The characteristic frequencies can be calculated by these lengths. For a given *P*, the length of the FFT area is *L* and the radius length of the diffraction peaks is *R*. According to the rule of FFT, $R/L = 1/\lambda = f/c$. All characteristic frequencies could be easily calculated.
